# Biological activity evaluations of chemical constituents derived from Mongolian medicinal forage plants and their applications in combating infectious diseases and addressing health problems in humans and livestock

**DOI:** 10.1007/s11418-021-01529-7

**Published:** 2021-05-21

**Authors:** Toshihiro Murata, Javzan Batkhuu

**Affiliations:** 1 Division of Pharmacognosy, Tohoku Medical and Pharmaceutical University, 4-1 Komatsushima 4-chome, Aoba-ku, Sendai, 981-8558 Japan; 2 School of Engineering and Applied Sciences, National University of Mongolia, POB-617/46A, Ulaanbaatar, 14201 Mongolia

**Keywords:** Mongolian, Medicinal, Forage, Native, Chemical, Constituents

## Abstract

Mongolian nomadic people possess traditional knowledge of wild plants that grow in their areas of habitation. Many of these are forage plants in nature and are consumed by livestock. However, these plants are known to have medicinal and/or toxic properties. To establish a scientific understanding of the plants, and in turn, offer sound knowledge on their applications and effective use, it is essential to collect data pertaining to the chemical constituents of each plant. Therefore, the first objective of this study was to identify and determine the structural constituents of the forage plants that were available to our research group. Furthermore, in an attempt to demonstrate the biological activities of the isolated chemical compounds, we focused on solving some of the social issues affecting Mongolian communities, including protozoan diseases affecting livestock, vectors of infectious diseases, and the general health of humans and their livestock. The results of the chemical constituents derived from Mongolian medicinal plants and their biological activities that were studied in the recent decade are also described herein.

**Graphic abstract:**

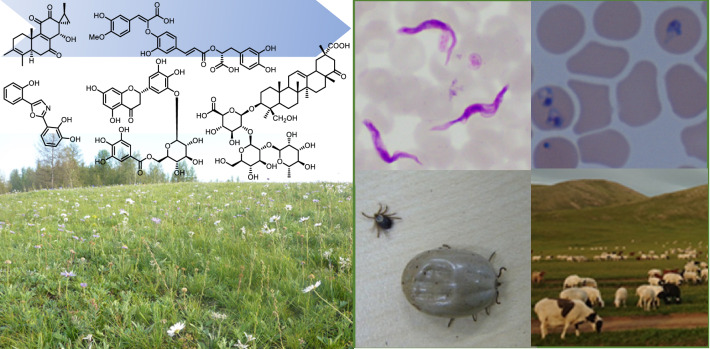

**Supplementary Information:**

The online version contains supplementary material available at 10.1007/s11418-021-01529-7.

## Introduction

A total of 3191 species of vascular plants belonging to 684 genera and 108 families account for the total plant population of Mongolia [1, 2]. Many of these plants are known to be useful to humans and livestock, with Mongolian people including nomads, appreciating the relationship between medicinal forage plants, and the well-being of humans and livestock. This knowledge has been passed on as a tradition from one generation to another. The Mongolian population uses medicinal prescriptions referred to as “jor” as well as medicinal herbs growing in the wild to prevent and cure many diseases. Mongolian traditional medicine is classified into the following categories, namely massage, acupuncture, herbal medicine, dietary cures, aromatherapy, phlebotomy, and sutra recitation. Herbal medicines are one of the principal remedies, which are thought to be derived from Tibetan traditional medicine [3, 4]. Although there are a few notable differences between the theories of herbal medicine and herbal sources of Mongolia and those of other countries, there are also some aspects in common. Herbal plant sources that are commonly used worldwide are abundant throughout Mongolia. These include the *Ephedra* herb and the *Glycyrrhiza* root [3], as well as the *Astragalus* root, *Scutellaria* root, *Bupleurum* root, *Saposhnikovia* root and rhizome [5], and peony root [6]. The aforementioned plants are important constituents of herbal drug formulations in various countries, including the Japanese Kampo formula. Descriptions about the sources of plants for Mongolian crude drugs are listed in the Mongolian national pharmacopeia published in 2011 (1st edition, Ministry of Health, Mongolia), and the animal drug pharmacopeia which is published by the Ministry of Food, Agriculture and Light Industry.

In addition to the plants used as raw materials for crude drugs, many continental plants that are freeze-proof and drought-tolerant are widely available in Mongolia [7], and the Gobi Desert [8]. These plants are distributed in severe environments with harsh conditions. The territory of this country is marked by diverse land with six natural vegetation zones from the north to the south: high mountain zone, taiga forest zone, mountain forest-steppe zone, steppe zone, desert steppe zone, and Gobi Desert zone [9]. In addition, a large variety of the continental climates in Mongolia result in rich biodiversity in each of the zones [10]. Many of the plants in this region have been known to possess chemical constituents that benefit humans, domestic livestock, and the surrounding ecosystem.

Some international and/or domestic groups have identified and characterized plants with medicinal benefits using scientific methods. The reports compiled from these investigations highlighted the features of the plants, including their distributions, usage, origins, medicinal components, and traditional history [7, 8, 11–14]. In addition, many researchers have studied the chemical constituents of wild plants in depth to determine their medicinal uses [15]. Nonetheless, due to the complexity of biological and ecological systems, frontier research continues to be relevant. Consequently, there is a need to investigate the biological activities of phytochemicals and their interactions with humans and animals to understand how these chemicals can be used more effectively.

Some of the significant plant resources of Mongolia are limited and have led to the publication of the endangered species list [2, 8, 16]. Although many researchers, including our own research group, have demonstrated fundamental aspects of various plants, further in-depth discussions are required on how to use these plants sustainably. Knowledge of the chemical constituents of each plant is fundamental data that is crucial in understanding the scientific features of the specific plant. The current research focused on the chemical constituents and biological activities of the Mongolian native plants and fungi, by investigating their traditional usage and collating information on their pharmacological effects and toxicity on animals.

### Attractive features of Mongolia, and the current problems that need attention

**Fig. 1:**
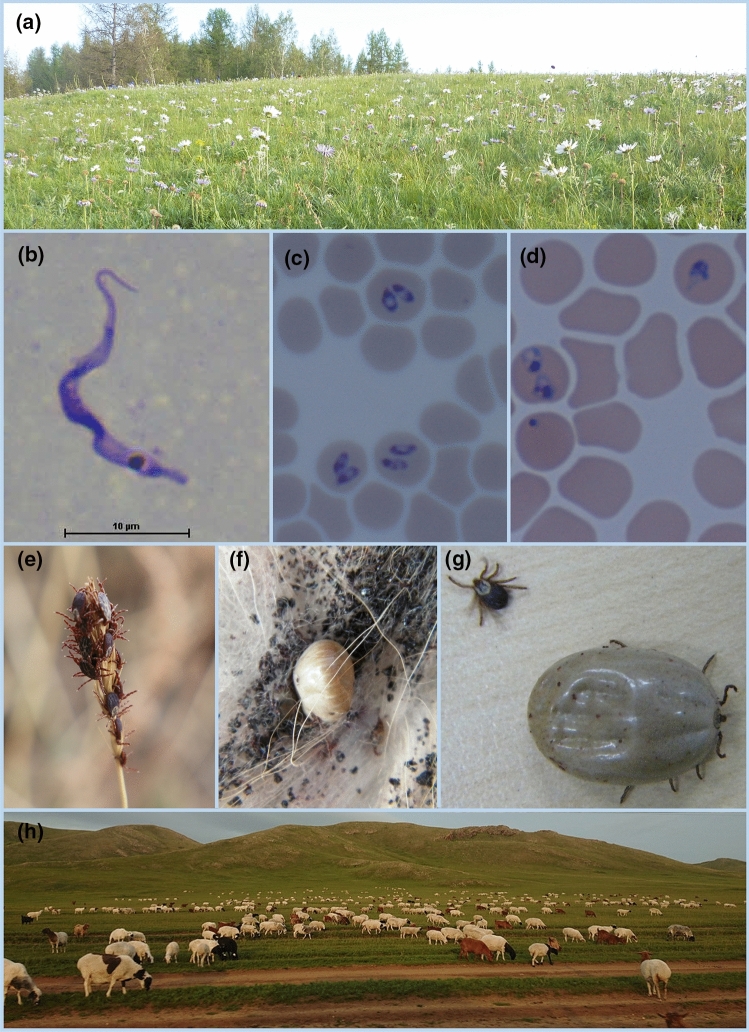
**a** Flower blooming plateau in Mongolia **b** *Trypanosoma equiperdum* (Photo by Dr. K. Suganuma), **c** *Babesia caballi* (Photo by Dr. B. Tuvshintulga), **d** *Theileria equi* (Photo by Dr. B. Tuvshintulga), **e** Ticks waiting for animals on a dried stem of a plant in early spring, **f** A tick sucking goat blood, **g** Ticks before and after sucking blood, **h** Livestock on the Mongolian steppe

The flower blooming plateau that extends beyond the horizon is recognized as one of the unique characteristic points of Mongolia (Fig. 1a). Over 20% of the population in Mongolia have adopted a nomadic lifestyle, with stock farming being one of the most important industries of the community. In 2016, the gross domestic product (GDP) ratio of the agricultural and livestock sectors accounted for 12.2% (second largest following the mining sector), which indicates the importance of the livestock sector in supporting the economy [17, 18]. Horses, cows, sheep, goats, and camels are among the five main livestock categories of the Mongolian community. In recent years, there have been over 60 million different heads of livestock in the Mongolian land (Fig. 1h) [17], and the latest report estimates around 70 million heads of animals [19]. The livestock is 20–25 times the population of the country. Although it is assumed that livestock graze freely, each animal has different palatability to forage plants of the Mongolian rangeland [20, 21].

Infectious diseases pose a threat while striving to attain a stable economy that depends on healthy human and livestock populations. In addition, livestock diseases and zoonosis are factors that contribute to the collapse of economies in nomadic communities. Protozoal diseases are caused by infectious protozoa, including *Trypanosoma*, *Babesia*, and *Theileria*. In Mongolia, *Trypanosoma equiperdum* infections in horses [22] and *Babesia* and *Theileria* infections in cattle [23] and horses [24] have been reported (Fig. 1b–d). Infected livestock become weak and lose their value. Moreover, severe natural disasters like “Dzud” cause the death of many of the weakened animals [25]. The *Babesia* and *Theileria* protozoans are mediated by ticks, including *Dermacentor nuttalli* [26]. Many ticks tend to parasitize thin animals after the winter season (Fig. 1e–g), resulting in health issues in these animals, such as loss of physical strength and reduced quality and quantity of its milk and meat. The bite marks and visual changes in the skin around these marks due to infestation by ticks, ultimately result in loss of potential value of the animal’s fur. Unfortunately, some of the animals are infected by pathogens that are tick-borne. Apart from ticks, unsanitary insects and small animals are also a cause for concern. Bacterial infectious diseases caused by *Yersinia pestis* and *Bacillus anthracis* as well as viral diseases have been reported. Although these diseases are rare, they are sometimes zoonotic and can cause fatality in the infected patients [27]. Infectious diseases are not confined to Mongolia alone, but are rather one of the widespread challenges that need worldwide attention [28]. New drugs that can eliminate pathogens are continuously being sought out, with strategies aiming to target the vector being one of the measures to prevent disease transmission. Nomads have been using many wild plants to cure infectious diseases, provide symptomatic relief, and remove epizoic insects and ticks of livestock since ancient times. Thus, it is important to study Mongolian plants to discover components with potential medicinal benefits against infectious diseases.

Apart from the effects on animals alluded to above, severe climates also have a direct and indirect effect on human life. Populations have concentrated in the urban areas because of the modernization of life, and it is feared that a potential dilution of the Mongolian traditions will occur due to a decrease in the nomadic community [29, 30]. Cold winters, dry summers, and strong sunshine over 1300 m above sea level cause problems such as skin conditions in native inhabitants. Recently, people in the urban areas have begun to worry about skin problems and allergies caused by various factors, including pollen.

Contrary to the fact that the population of nomads is decreasing because of economic development, livestock farming has continued to increase in Mongolia [29]. Plant resources have depleted in these areas due to excessive livestock farming. Rangelands that previously had various plant species growing in them have now become areas with plentiful growth of only *Artemisia* plants [31]. Moreover, some of these areas became desertified, which in turn exacerbated the difficulty in maintaining the quality of meat and milk of livestock. Furthermore, some countries prohibit the sale of Mongolian meat because the concentrations of antibiotics in the meat appear to exceed reference values. To solve these problems, a scientific approach to study the chemical interactions between forage plants and livestock is essential.

### Our approach to solving the problems using plants and their chemical constituents

Our research objective proposed to establish solutions to the above-mentioned problems using Mongolian plant resources and their chemical constituents. During the last decade, in a collaborative research project, a total of 373 chemical compounds (including over 110 new natural products) were identified from plants and fungi in Mongolia (Table S1, Supplementary Information). These compounds included alkaloids, flavonoids, phenylpropanoids, lipids, terpenoids, saponins, glycosides, and their derivatives. An in-depth evaluation of the isolated compounds to determine their pharmaceutical properties was conducted in an attempt to solve the above-mentioned problems experienced by the Mongolian community [32–55].

### Trypanocidal activity

**Fig. 2:**
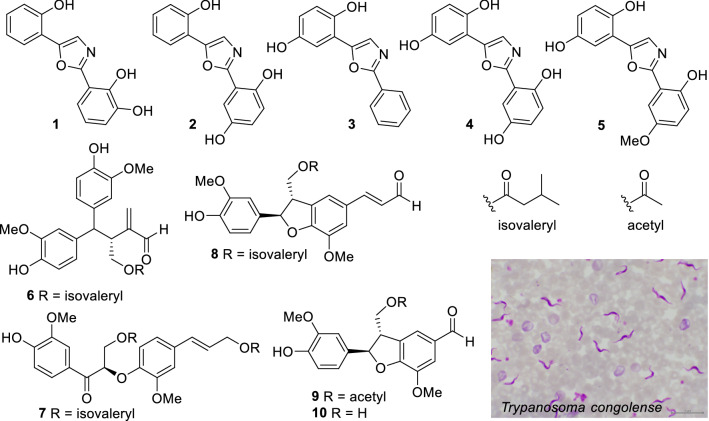
Oxazoles isolated from *Oxytropis lanata* (**1**–**5**) and lignans from *Brachanthemum gobicum* (**6**–**10**) possess trypanocidal activity, and *Trypanosoma congolense* (Photo by Dr. K. Suganuma)

In a collaborative research project conducted along with a veterinary group, various types of antiprotozoal compounds were isolated and identified from Mongolian medicinal plants. *Oxytropis lanata* is a perennial herb belonging to the family Fabaceae. *Oxytropis lanata* is used to treat fractures, wounds, and reduce inflammation. From this plant, eleven 2,5-diphenyloxazoles were isolated together with six known isoflavonoid derivatives. Oxazole-type alkaloids are rare natural products, but the oxazole structure is a fundamental moiety in certain pharmaceutical and drug candidate compounds. In the screening for trypanocidal activity against *Trypanosoma congolense* which causes the lethal disease “nagana” in animals, compound **1** (Fig. 2) showed the highest activity (IC_50_ 1.0 μM) while compounds **2**–**5** (Fig. 2) had potent activities (IC_50_ 6.0–14.8 μM) [32]. In addition, lignans also presented as potent anti-*Trypanosomal* candidates. Characteristic acylated lignans were isolated from *Brachanthemum gobicum* (Asteraceae). They were isolated as pairs of enantiomers and their absolute configurations were determined from their optical rotations and electronic circular dichroism (ECD) spectra [33]. These *Brachanthemum* phenylpropanoid dimers (**6–10**, Fig. 2), sesamin from *Artemisia sieversiana* (Asteraceae), and some flavonoids from the above plants showed trypanocidal activity (IC_50_ 2.4–10.0 μM) [32, 34, 35]. Naranmandakh and coworkers also demonstrated lanostane triterpenoids, isolated from a fungus *Fomitopsis officinalis* in the family Polyporaceae [36], as active compounds. *Trypanosoma congolense* is transmitted by tsetse flies and causes severe animal diseases in African countries; however, this species is not widely distributed throughout Mongolia. Nevertheless, Suganuma and coworkers isolated and characterized the Mongolian *Trypanosoma equiperdum* strain that causes “dourine” [22]. Testing of the above active compounds to study their pharmaceutical effects on Mongolian *T*. *equiperdum* is expected to be undertaken as the next project because in vitro assay systems for this strain have been already established [37].

### Anti-piroplasm activity

**Fig. 3:**
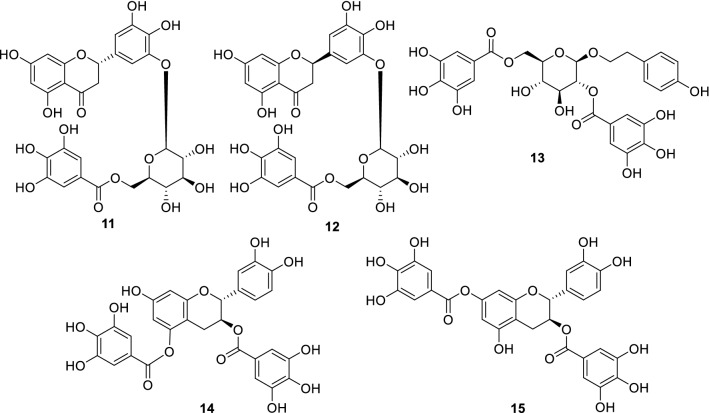
Anti-piroplasma compounds isolated from *Saxifraga spinulosa* (**11**–**13**) and *Bergenia crassifolia* (**14** and **15**)

Piroplasmosis is caused by pathogenic protozoa of the genera *Babesia* and *Theileria*. *Babesia bovis* and *B*. *bigemina* infect cattle while equine piroplasmosis is caused by *B*. *caballi* and *Theileria equi*. These protozoal diseases cause hemolytic anemia, fever, hemoglobinuria, and splenomegaly in animals and negatively affect animal husbandry [23, 24, 38]. *Saxifraga spinulosa* belongs to the family Saxifragaceae and is known to be consumed by elks [20]. Badral and coworkers reported isolating galloyl glycosides, including flavonoids that have a glucosyl moiety connected to the C-3′ carbon of a pyrogallol B-ring, a rare structural feature, from this plant [38]. Although there were differences in sensitivity, the isolated compounds from this plant showed anti-piroplasm activity against some of the four strains described above. For example, new compounds **11** and **12** (Fig. 3) showed inhibitory activities against *Babesia bovis* and *B*. *bigemina* (IC_50_ 9.4 ~ 22.7 μg/mL), and compound **13** showed inhibitory activity against *B*. *caballi* (IC_50_ 15.6 μg/mL). The galloyl group is a key structure for anti-piroplasm activity, with experiments of flavonoids isolated from *Bergenia crassifolia* of the family Saxifragaceae (**14** and **15**, Fig. 3), supporting this conclusion [39]. Nonetheless, some flavan-3-ols without the galloyl group also showed moderate inhibitory activity against *Babesia* and *Theileria* [40].

### Virucidal and antibacterial activity

**Fig. 4:**
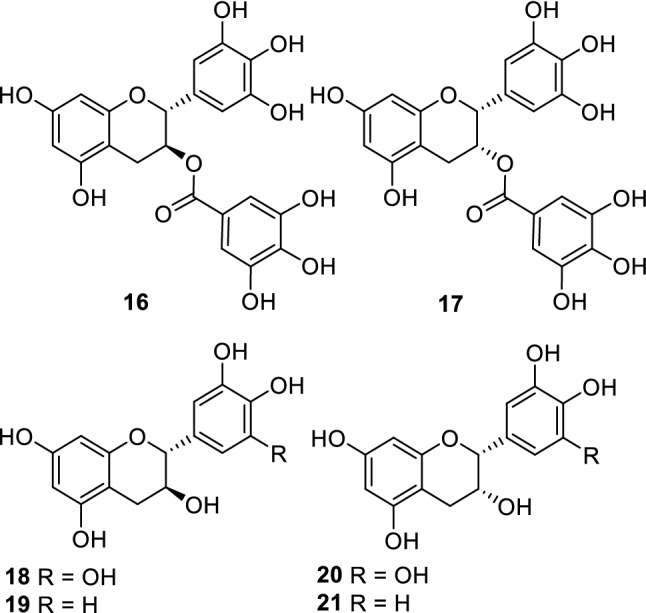
Catechin and its analogues (**16**–**21**)

Currently, humanity is facing a global catastrophe as the infectious disease Covid-19, which is caused by the severe acute respiratory syndrome coronavirus 2 (SARS-CoV-2), continues to spread. One of the ways to combat the spread of Covid-19 is to inactivate the SARS-CoV-2. Takeda and coworkers conducted virucidal screening against multiple viruses, using a library of extracts and compounds that was available to the group. In this study, fractions and some compounds, including gallocatechin gallate (GCG) (**16,** Fig. 4) and epigallocatechin gallate (EGCG) (**17,** Fig. 4), from *Saxifraga spinulosa* showed potent activity against the influenza A virus, feline calicivirus, murine norovirus, and SARS-CoV-2 [41]. The effects of EGCG have been investigated by many groups because it is a typical tea catechin. However, the crude fractions of GCG and EGCG showed higher activities than GCG and EGCG. We propose the use of a more refined approach in the future to identify more effective components.

**Fig. 5:**
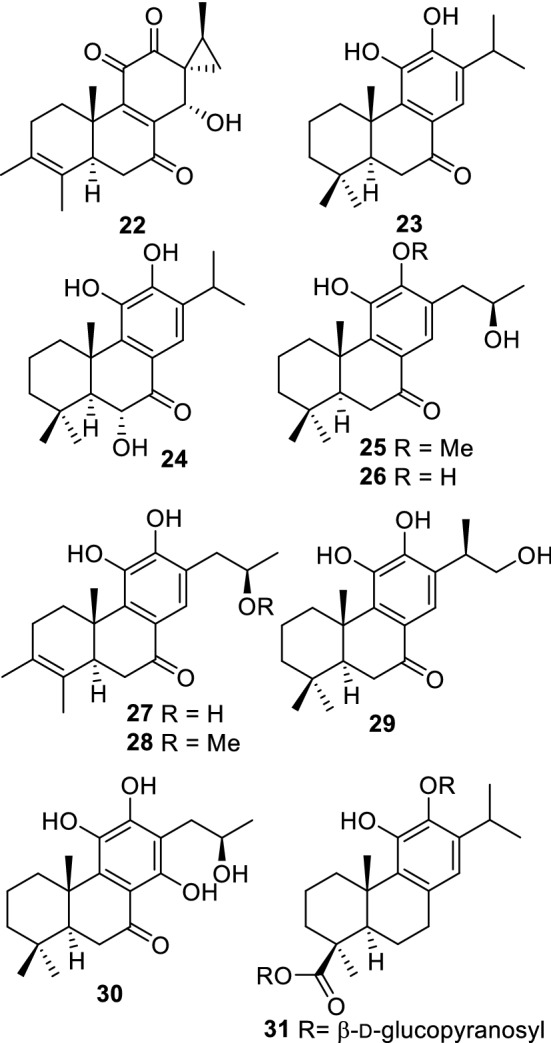
Diterpenoids (**22**–**31**) isolated from *Caryopteris mongolica* possess antibacterial and cholinesterase inhibitory activities

The search and isolation of new antibiotics continue to be a vital area of focus in research because of emerging drug-resistant bacterial strains. *Caryopteris mongolica*, a plant belonging to the family Lamiaceae, is moderately palatable to goats and horses [20]. Saruul and coworkers isolated a diterpenoid (**22**, Fig. 5) which has *ortho*-quinone and spiro-methylcyclopropane moieties from *Caryopteris mongolica*. The compound showed antibacterial activity against Gram-positive bacteria: *Staphylococcus aureus*, *S*. *epidermidis*, *Enterococcus faecalis*, and *Micrococcus lutens* [42]. In addition, extracts and fractions from *Comarum salesovianum* (Rosaceae) showed inhibitory activity against the aforementioned strains. Consequently, the constituents of this plant were investigated by Odontuya and coworkers, and phenolic lipid derivatives possessing antibacterial properties were identified [43].

### Vectors of pathogens

Various biting ticks and insects, such as lice, are known vectors of pathogens. These vectors are responsible for transmitting a number of protozoal, bacterial, and viral infectious diseases in humans and animals. Vector control is, therefore, one of the most important ways to prevent the transmission of these infectious diseases.

*Dermacentor nuttalli* is a tick known as one of the major unsanitary pests in Mongolia, and mediates infectious diseases between livestock. Banzragchgarav and coworkers examined 113 crude extracts of 76 plant species included in 26 families for anti-tick activity against *D*. *nuttalli*. The extracts from *Amaranthus retroflexus* (Amaranthaceae) roots, *Ephedra sinica* (Ephedraceae) leaves, *Erigeron acer* (Asteraceae) roots, *Ranunculus japonicus* (Ranunculaceae) roots, and *Spiraea salicifolia* (Rosaceae) leaves showed inhibitory activity against *D*. *nuttalli* [44].

The analyses of the active compounds and mechanisms of inhibition of the above-mentioned anti-tick plants are still ongoing. In addition, in an attempt to identify any plant-vector (insect, tick, and lice) chemical interaction(s), we tried to estimate the effect(s) of phytochemicals in an insect model. Phenoloxidase is a component of the innate immune system of insects, which defends against foreign substances by producing melanin. In our study, an assay was developed for phenoloxidase activity using *Acyrthosiphon pisum*. The inhibitory effect against phenoloxidase of compounds from *Atraphaxis frutescens* (Polygonaceae) was estimated by Odonbayar and coworkers, by comparing the results with the 1,1-diphenyl-2-picrylhydrazyl (DPPH) radical scavenging activities and mushroom tyrosinase activities [45]. In the above study, gallocatechin (**18,** Fig. 4) and epigallocatechin (**20,** Fig. 4) were suggested to be key compounds that inhibit phenoloxidase. Furthermore, the activity of catechin (**19,** Fig. 4) was ten times higher than that of epicatechin (**20**, Fig. 4), which was confirmed by the experiments that were conducted using pure samples during an investigation of the chemical constituents from *Calligonum mongolicum* of the family Polygonaceae [46]. *Brachanthemum gobicum* is used to remove external parasites, such as the parasitic louse *Linognathus*, from domesticated sheep [33]. The characteristic acylated lignans have an isovaleryl moiety, which is released by the disassembly of the compounds. Therefore, the effect of the lignans against parasites is of interest.

### Effects on human and animal health

**Fig. 6:**
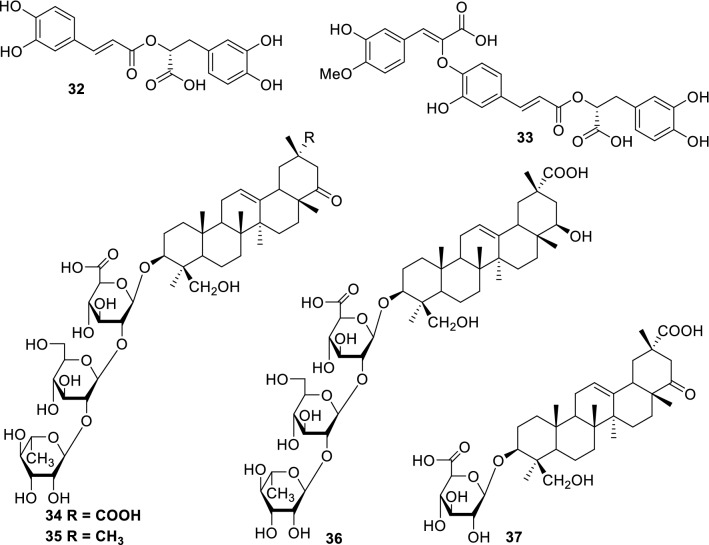
Phenylpropanoid oligomers isolated from *Dracocephalum foetidum* (**32** and **33**) and saponins (**34**–**37**) from *Oxytropis lanata* possess hyaluronidase inhibitory activity

Hyaluronic acid (HA) is one of the main elements of the body’s extracellular matrix and has numerous functions in the skin, joints, and internal organs. Hyaluronidase is an enzyme that degrades HA. Inhibitors of hyaluronidase are expected to control the amount of HA, and inhibition activities of this enzyme have been hypothesized to relate to anti-allergic activity [56, 57]. *Dracocephalum foetidum* (Lamiaceae) is a herbal plant used to treat inflammatory conditions. Selenge and coworkers identified the hyaluronidase inhibitory phenylpropanoid oligomers, rosmarinic acid (**32**, Fig. 6), its derivative (**33**, Fig. 6), and flavonoids as chemical constituents of this plant [47]. Compounds showing hyaluronidase inhibitory activity were also found in plants belonging to the family Rosaceae, including *Chamaerhodos erecta*, *C*. *altaica*, and *Dasiphora parvifolia*. These compounds were identified as hydrolysable tannins, dimeric A-type proanthocyanidins, and catechins [48, 49]. Furthermore, various triterpene saponins have been reported as hyaluronidase inhibitors, with some of the active saponins that have oleanene-type triterpene aglycone (**34–37**, Fig. 6) being isolated from aerial parts of *Oxytropis lanata* by Buyankhishig and coworkers [34].

**Fig. 7:**
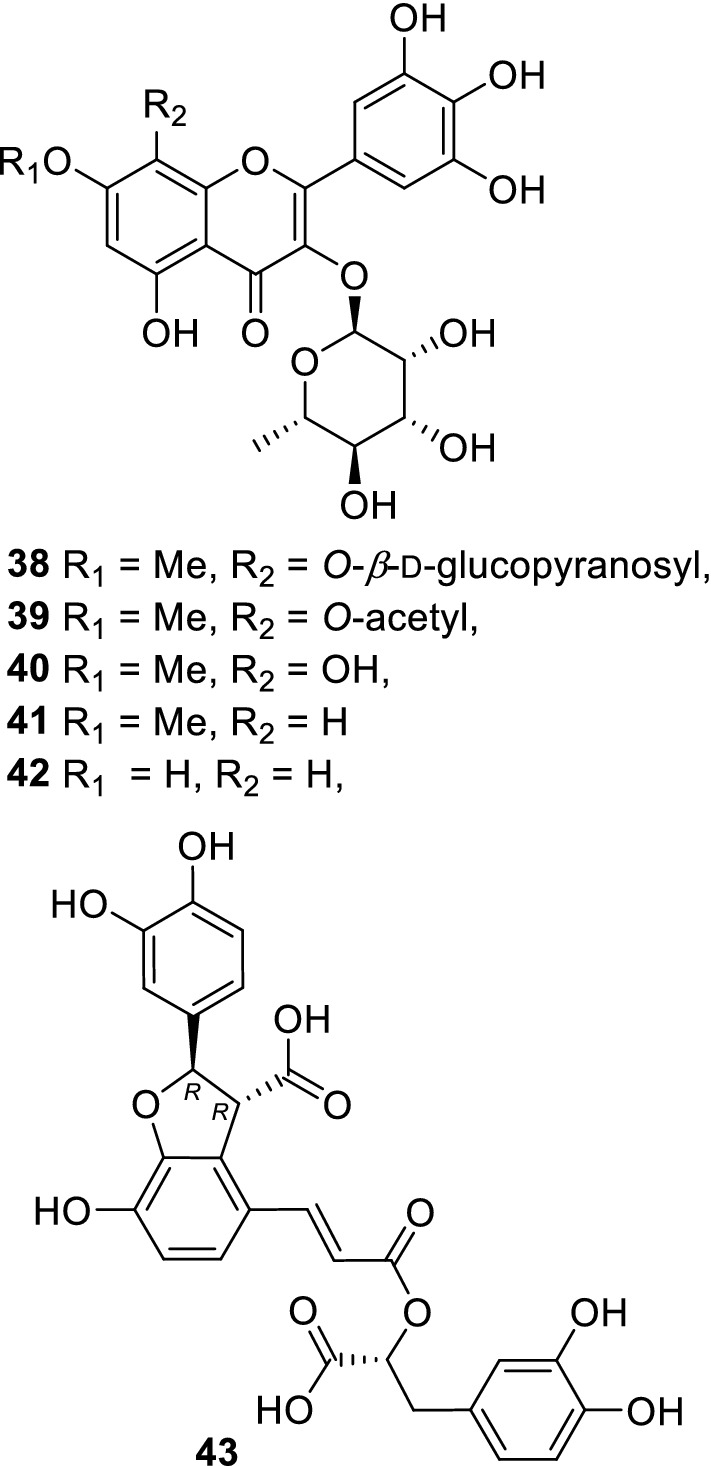
Flavonoid glycosides isolated from *Atraphaxis frutescens* (**38**–**42**) possess tyrosinase inhibitory activity and monardic acid A (**43**) isolated from *Thymus gobicus* possesses cholinesterase inhibitory activity

It is understood that antioxidants affect oxidative steps and generally decrease oxidative stresses in the body. DPPH radical scavenging activity is considered to be one of the indexes of antioxidant activity. Therefore, the radical scavenging activity of isolated compounds was determined [38, 45, 47–50]. Melanin, a skin pigment, is produced by the oxidation of amino acids, including tyrosine. Consequently, tyrosinase is an enzyme of concern during melanization, with some of the isolated anti-oxidative compounds showing tyrosinase inhibitory activity. Compounds containing 7-methoxyflavonols with pyrogallol B-ring moieties and myricitrin (**38**–**42**, Fig. 7), isolated from *Atraphaxis frutescens,* showed inhibitory activity against mushroom tyrosinase (IC_50_ 0.9–4.7 mM) [45]. Advanced glycation end products (AGEs) are considered to induce and promote degenerative diseases, including diabetes and Alzheimer’s. AGEs are produced from carbohydrates, amino acids, and proteins via various steps including oxidation. We evaluated the inhibitory activity of AGEs, and many constituents isolated from *Chamaerhodos erecta* and *C*. *altaica* showed activity [48]. Triterpenes having a γ-lactone ring were obtained from *Abies sibirica,* which belongs to the Pinaceae family. Five of these triterpenes showed low-density lipoprotein (LDL)-antioxidative activity, and some of the triterpenes showed lipase inhibitory activity [51]. Livestock palatability studies for plants containing anti-oxidative compounds have been reported. It was noted that camels prefer *Atraphaxis frutescens* during the summer, autumn, and winter seasons. This plant is thought to be an important component of mixed livestock feed because of its high nutrient values [20]. In addition, *Chamaerhodos altaica* is known to be a good forage plant for horses and small animals [20] while *Dracocephalum foetidum* is moderately consumed by camels in the summer season [20, 21]. The next theme of our research work aims to focus on the chemical interactions of the identified plant compounds with livestock.

Cholinesterase is an enzyme regulating the levels of acetylcholine in various tissues of the body. Acetylcholinesterase (AChE) and butyrylcholinesterase (BChE) are largely involved in the neural transmission of synapses and their inhibitors have been identified as targets in the treatment of neurodegenerative disorders, including Alzheimer’s diseases and myasthenia gravis [58]. Abietane-type diterpenoids (**23–30**, Fig. 5) from *Caryopteris mongolica* showed AChE and/or BChE inhibitory activity [52, 53]. Among these, compound **24** showed the highest activity (AChE from human erythrocytes: IC_50_ 19.2 μM; AChE from electric eel: IC_50_ 12.3 μM; BChE from horse serum: IC_50_ 7.70 μM). Although a new diterpenoid glucoside (**31**) was isolated, it did not show AChE or BChE inhibitory activity [52]. The diterpenoids have catechol moieties, and the absolute configuration of compound **30** was determined by X-ray crystallography [53]. Furthermore, some types of phenylpropanoid oligomers showed AChE and BChE inhibitory activity. *Thymus gobicus* (Lamiaceae) contained AChE and BChE inhibitory monardic acid A (**43**, Fig. 7) [54], which had the 7*R*,8*R*-configuration instead of the 7*S*,8*S*-configuration of lithospermic acids as a structural feature [59].

### Chemical interactions between forage plants and livestock

*Pulsatilla* genus plants are termed “Yargui” in Mongolia. The flowers of Yargui are traditionally welcomed by nomads because they are regarded as a tonic for livestock that has survived the harsh winter, specifically, goats and sheep [20, 21]. Moreover, consuming the meat and milk of livestock that has fed on early spring Yargui flowers is seen as a promise of a healthy life for the year by the nomadic people [40]. *Pulsatilla flavescens* (Ranunculaceae) is a perennial herb commonly called yellow Yargui by the nomadic people. Ganchimeg and coworkers investigated the constituents of the flower, and obtained numerous flavonoids [40]. Although the reasons behind the tonic effects have not been established, we will approach the important traditions of nomadic people and their livestock from a scientific perspective as a next step.

**Fig. 8:**
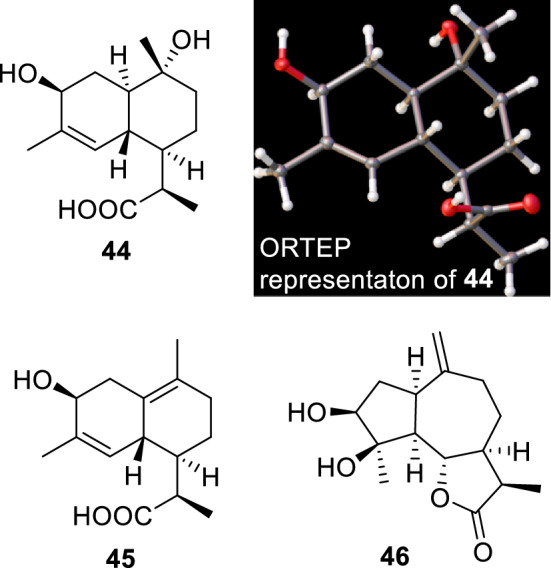
Sesquiterpenoids isolated from *Artemisia sieversiana* (**44**–**46**)

*Artemisia sieversiana* is an annual herbaceous plant named “tsarvant sharilj.” Its flower is used to treat fever, throat inflammation, and pneumonia in Mongolia. Although the plant is thought to be a forage plant, livestock preferences for the plant differ by season [20, 21]. For example, it is most palatable to camels, goats, and sheep in spring but is not consumed by the livestock during summer. Although the livestock consume withered *A*. *sieversiana* after autumn, nomadic people have noted the production of meat and milk with an unpleasant odor and flavor during this period. In an attempt to identify the chemical constituents of this plant, Nurbek and coworkers isolated sesquiterpenoids (**44**–**46**, Fig. 8), dimers of sesquiterpenoids, flavonoids, lignans, and quinic acid derivatives [35]. The absolute configuration of compound **44** was established using single-crystal X-ray diffraction, and the isolated sesquiterpenoids from Mongolian *A*. *sieversiana* were different from known isomers [35]. Although the main constituents of this plant have been identified, the reason(s) as to why the plant has undesirable effects on the meat and milk of livestock after autumn still needs to be established.

### Future development

Research on the structure–activity relationship of natural and synthesized compounds to identify compounds targeting *Trypanosoma* species and various pathogens has already started. To control infectious diseases, it is important to study not only pathogens but also their vectors; hence, we are investigating the chemical interactions involved in their biological mechanisms. Cholinesterase is a target for pesticides, and selective acetylcholine inhibitors have been used to produce insecticides. In our experiments, although the effects of plant compounds on human and animal cholinesterase were evaluated [52–54], their selectivity against animal and pest species including the detailed mechanisms of their effects on enzymes have to be considered. Furthermore, the effects of plant-derived compounds on the innate immune system of insects and ticks are interesting themes for future research [45, 46].

It is generally thought that many plant metabolites are produced by evolutionary (and sometimes coevolutionary) mechanisms based on the results of interactions between plants and insects, animals, microorganisms, and environmental conditions. Plants have been shown to influence the health of animals through their chemical constituents. For example, some of the genus *Oxytropis* plants such as *Oxytropis glabra* are known as toxic plants because they contain an indolizidine alkaloid, swainsonine, which causes lethal neurological disorders [60]. However, although there is no knowledge on the toxic constituent(s) of *Oxytropis lanata*, the biologically active oxazoles and saponins can be beneficial [32, 34]. Furthermore, even though *O*. *lanata* is not palatable in pasture lands [21], it would be of interest to know whether these plants are good forage or medicinal plants. Research on their phytochemicals would be able to provide further information.

*Brachanthemum gobicum* have isovaleryl moieties in lignans [33], and isovaleric acid is easily released by their hydrolysis. Although *B*. *gobicum* are known as desirable forage plants for camels in spring and autumn [20, 21], humans perceive isovaleric acid as having a strong unpleasant odor. An area of interest would be the effect of isovaleric acid on animals, including plant palatability and repellent properties. For goats and sheep, smell and taste are important senses that are used to judge edible plants. In addition, it has been shown that herbivorous insects are stimulated to feed by nutritional and plant secondary components [61]. However, the compounds and tastes that stimulate the foraging behaviors of herbivorous livestock are yet to be determined [62]. Livestock in Mongolia rotate their feeding fields depending on the seasons and environmental conditions. They tend to judge and know nutritious or toxic plants of the flora that keeps changing every season.

Available scientific data are currently not enough to understand the interactions in complex ecosystems. However, continuous research based on chemical, biological, and ecological approaches to study the interactions between livestock and wild plants will present new ideas on how to treat severe infectious diseases in both humans and livestock.

## Conclusions

The characteristic chemical constituents from Mongolian medicinally useful plants were isolated and their biological activities were studied. For example, 2,5-diphenyloxazoles isolated from *Oxytropis lanata* and acylated-lignans isolated from *Brachanthemum gobicum* are Trypanocidal compounds. Galloyl moieties are expected to be one of the key structures showing anti-piroplasm activities as postulated from the results of the research on plants belonging to the family Saxifragaceae. The phytochemicals that can potentially prevent vector problems were investigated. Some plant extracts and phenoloxidase inhibitory compounds were found to be potential inhibitors of *Dermacentor nuttalli* and unsanitary insects. Flavonoids, phenylpropanoid derivatives, and phenolic compounds from *Atraphaxis frutescens* and some *Dracocephalum* plants showed hyaluronidase inhibitory and/or radical scavenging activity. Catechins and their analogs were isolated from many plants during our study and they may have key roles in exerting pharmacological and biological effects. They may be useful against stressful lifestyles and can thus, contribute to the care and well-being of humans and animals.

There are many potent plant sources in the nomadic habitats of Mongolia and significant traditional knowledge possessed by this population. These attributes need to be studied in depth to determine their suitability for sustainable application. The chemical constituents of these plants are an informative foundation for the scientific understanding of the ecology related to nomadism, which may provide numerous ideas for better and healthier human lives.

## Supplementary Information

Below is the link to the electronic supplementary material.

Supplementary file1 (PDF 912 kb)
