# Supplementary material for: Biological activity evaluations of chemical constituents derived from Mongolian medicinal forage plants and their applications in combating infectious diseases and addressing health problems in humans and livestock
Source: J Nat Med. 2021 May 21;75(4):729–40. doi: 10.1007/s11418-021-01529-7 (PMC8137442; doi:10.1007/s11418-021-01529-7)
Supplement: Supplementary file 1 — Supplementary file1 (PDF 912 kb) [file 11418_2021_1529_MOESM1_ESM.pdf]

Supplementary Information for **Biological activity evaluations of chemical constituents derived from Mongolian medicinal forage plants and their applications in combating infectious diseases and addressing health problems in humans and livestock**

Journal of Natural Medicines, DOI: https://doi.org/10.1007/s11418-021-01529-7

Toshihiro Murata[1,*], Javzan Batkhuu[2]

Affiliations

[1] Tohoku Medical and Pharmaceutical University, Sendai, Japan

[2] National University of Mongolia, Ulaanbaatar, Mongolia

*Corresponding author.

Dr. MURATA Toshihiro

Tel: +81 22 727 0086. Fax: +81 22 727 0220.

E-mail address: murata-t@tohoku-mpu.ac.jp

Table of contents

Table S1 Isolated compounds from Mongolian plants and fungi and their biological activities········ S1~S13

Table S1 Isolated compounds from Mongolian plants and fungi and their biological activities

plants and fungi family, genus, and species	parts	chemical name	biological activity	IC_{50} value[a]	reference number	compound number
Asteraceae						
Artemisia sieversiana Ehrh. ex Willd.	aerial parts	tsarvanin A			[35]	**44**
		tsarvanin B			[35]	**45**
		tsarvanin C			[35]	**46**
		costuslactone B			[35]	
		artabsinolide A			[35]	
		absinthin			[35]	
		anabsinthin			[35]	
		absinthin A			[35]	
		chrysoeriol	inhibition against *Trypanosoma congolense*	16.5 μM	[35]	
		tricin	inhibition against *Trypanosoma congolense*	90.2 μM	[35]	
		chrysosplenetin	inhibition against *Trypanosoma congolense*	2.9 μM	[35]	
		spinacetin 3-*O*-rutinoside			[35]	
		epiyangambin	inhibition against *Trypanosoma congolense*	17.2 μM	[35]	
		de-*O*-methylepimagnolin A			[35]	
		yangambin			[35]	
		diayangambin			[35]	
		sesamin	inhibition against *Trypanosoma congolense*	10.0 μM	[35]	
		epiaschantin			[35]	
		spinacetin 3-*O*-β-D-glucopyranoside			[35]	
		kaempferol 3-*O*-β-D-glucopyranoside			[35]	
		chlorogenic acid			[35]	
		1,5-dicaffeoylquinic acid			[35]	
		1,5-dicaffeoylquinic acid methyl ester			[35]	
Brachanthemum gobicum Krasch.	aerial parts	brachangobinan A	inhibition against *Trypanosoma congolense*	13.4 μM	[33]	
		(7'*R*,8'*S*)-brachangobinan A			[33]	
		(7'*S*,8'*R*)-brachangobinan A			[33]	
		brachangobinan B	inhibition against *Trypanosoma congolense*	2.8 μM	[33]	**6**
		(8*R*)-brachangobinan B			[33]	
		(8*S*)-brachangobinan B			[33]	
		brachangobinan C	inhibition against *Trypanosoma congolense*	17.4 μM	[33]	**7**
		(7'*R*,8'*R*)-brachangobinan C			[33]	
		(7'*S*,8'*S*)-brachangobinan C			[33]	
		brachangobinan D	inhibition against *Trypanosoma congolense*	9.6 μM	[33]	
		brachangobinan E			[33]	
		(7*R*,8*R*)-brachangobinan E			[33]	
		(7*S*,8*S*)-brachangobinan E			[33]	
		brachangobinan F			[33]	
		brachangobinan G			[33]	
		brachangobinan H	inhibition against *Trypanosoma congolense*	2.4 μM	[33]	**8**
		(7'*R*,8'*S*)-brachangobinan H			[33]	
		(7'*S*,8'*R*)-brachangobinan H			[33]	
		brachangobinan I	inhibition against *Trypanosoma congolense*	19.9 μM	[33]	**9**
		(7'*R*,8'*S*)-brachangobinan I			[33]	

		(7'*S*,8'*R*)-brachangobinan I			[33]	
		brachangobinan J			[33]	
		brachangobinan K			[33]	
		dimeric coniferyl acetate	inhibition against *Trypanosoma congolense*	16.2 μM	[33]	
		3-acetoxymethyl-5-[(*E*)-2-formylethen-1-yl]-2-(4-hydroxy-3-methoxyphenyl)-7-methoxy-2,3-dihydrobenzofuran	inhibition against *Trypanosoma congolense*	7.6 μM	[33]	
		balanophonin	inhibition against *Trypanosoma congolense*	9.5 μM	[33]	**10**
		coniferyl isovalerate			[33]	
		trans-ferulic acid			[33]	
		4-hydroxy-3-methoxycinnamaldehyde			[33]	
		vanillin			[33]	
Fabaceae						
Oxytropis lanata (Pall.) DC.	aerial parts	3-*O*-[α-L-rhamnopyranosyl(1→2)-β-D-glucopyranosyl(1→2)-β-D-glucuronopyranosyl]-[3'-hydroxy-2'-methyl-5',6'-dihydro-4'-pyrone(6'→)]-3β,22β,24-trihydroxyolean-12-ene			[34]	
		3-*O*-[β-D-glucopyranosyl(1→2)-β-D-glucuronopyranosyl]-3β,22β,24-trihydroxyolean-12-en-29-oic acid			[34]	
		3-*O*-[α-L-rhamnopyranosyl(1→2)-β-D-glucopyranosyl(1→2)-β-D-glucuronopyranosyl]-3β,24-dihydroxyolean-12-en-22-oxo-29-oic acid	hyaluronidase inhibitory	150 μM	[34]	**34**
		azukisaponin V			[34]	
		robinoside B	hyaluronidase inhibitory	210 μM	[34]	**36**
		3-*O*-[β-D-glucopyranosyl-(1''-2')-β-D-glucuronopyranosyl] soyasapogenol B			[34]	
		robinoside F			[34]	
		caraganin B	hyaluronidase inhibitory	220 μM	[34]	**37**
		dehydroazukisaponin V	hyaluronidase inhibitory	150 μM	[34]	**35**
		kaempferol			[34]	
		kaempferol 3-*O*-β-D-glucopyranoside			[34]	
		quercetin 3-*O*-β-D-glucopyranoside			[34]	
		quercetin 3-*O*-β-D-glucuronide			[34]	
		isorhamnetin 3-*O*-β-D-glucuronide			[34]	
		quercetin 3-*O*-β-D-glucuronide-6''-methyl ester			[34]	
		isorhamnetin 3-*O*-β-D-glucuronide-6''-methyl ester			[34]	
		kaempferol 3-*O*-(6''-*O*-malonyl)-β-D-glucopyranoside			[34]	
		quercetin 3-*O*-(6''-O-malonyl)-β-D-glucopyranoside			[34]	
		isorhamnetin 3-*O*-(6''-*O*-malonyl)-β-D-glucopyranoside			[34]	
		kaempferol 3-rutinoside			[34]	
		quercetin 3-rutinoside			[34]	
		isorhamnetin 3-rutinoside			[34]	
		naringenin 4'-*O*-β-D-glucopyranoside			[34]	
		dihydrokaempferol 4'-*O*-β-D-glucopyranoside			[34]	
		5,7,4'-trihydroxy isoflavone	inhibition against *Trypanosoma congolense*	10.5 μM	[34]	
		genistein-7-yl β-D-glucopyranoside			[34]	
		6''-*O*-malonylgenistin			[34]	
		arizonicanol A	inhibition against *Trypanosoma congolense*	4.1 μM	[34]	
		3,9-dimethyl-10-hydroxy-pterocarpan			[34]	
		isophentyl β-D-glucopyranoside			[34]	
		benzyl alcohol β-D-glucopyranoside			[34]	
		2-phenylethyl β-D-glucopyranoside			[34]	
		2-(3'-hydroxyphenyl)-5-(2''-hydroxyphenyl)-oxazole			[34]	
		2-(2',5'-dihydroxyphenyl)-5-(2''-hydroxyphenyl)-oxazole	inhibition against *Trypanosoma congolense*	12.2 μM	[34]	
		(+)-(9*Z*,11*E*,13*S*,15*Z*)-octadeca-9,11,15-trien-13-olide			[34]	

			tryptophan			[34]	
	Oxytropis lanata (Pall.) DC.	roots	2,5-diphenyloxazole			[32]	
			2-phenyl-5-(2''-hydroxyphenyl) oxazole			[32]	
			2-(3'-hydroxyphenyl)-5-(2''-hydroxyphenyl) oxazole			[32]	
			2-(2',3'-dihydroxyphenyl)-5-(2''-hydroxyphenyl) oxazole	inhibition against *Trypanosoma congolense*	1.0 μM	[32]	**1**
			2-(2',5'-dihydroxyphenyl)-5-(2''-hydroxyphenyl) oxazole	inhibition against *Trypanosoma congolense*	12.2 μM	[32]	**2**
			2-phenyl-5-(2'',5''-dihydroxyphenyl) oxazole	inhibition against *Trypanosoma congolense*	12.1 μM	[32]	**3**
			2-(2',5'-dihydroxyphenyl)-5-(2'',5''-dihydroxyphenyl) oxazole	inhibition against *Trypanosoma congolense*	14.8 μM	[32]	**4**
			2-(2'-hydroxy-5'-*O*-methylphenyl)-5-(2'',5''-dihydroxyphenyl) oxazole	inhibition against *Trypanosoma congolense*	6.0 μM	[32]	**5**
			2-(2',5'-dihydroxyphenyl)-5-phenyl oxazole			[32]	
			3,9-dimethyl-10-hydroxypterocarpan			[32]	
			vesticarpan			[32]	
			(3*R*)-(−)-arizonicanol A	inhibition against *Trypanosoma congolense*	4.0 μM	[32]	
			5'-methylvestitol			[32]	
			odoratin			[32]	
			afromosin			[32]	
Lamiaceae							
	Caryopteris mongolica Bunge	aerial parts	12,19-di-*O*-β-D-glucopyranosyl-11-hydroxyabieta-8,11,13-triene-19-one			[52]	**31**
			isoscutellarein 7-*O*-[β-D-xylopyranosyl-(1→2)]-β-D-glucopyranoside			[52]	
			isoscutellarein 7-*O*-[α-L-rhamnopyranosyl-(1→2)]-β-D-glucopyranoside			[52]	
			hypolaetin 7-*O*-[6''-*O*-(*p*-*E*-coumaroyl)]-β-D-glucopyranoside			[52]	
			hypolaetin 7-*O*-[6''-*O*-(*E*-caffeoyl)]-β-D-glucopyranoside			[52]	
			hypolaetin 7-*O*-β-D-glucopyranoside			[52]	
			luteolin			[52]	
			acacetin			[52]	
			hypolaetin			[52]	
			luteolin 7-*O*-[6''-*O*-(*trans*-caffeoyl)]-β-D-glucopyranoside			[52]	
			luteolin 7-*O*-[6''-*O*-(*trans*-feruloyl)]-β-D-glucopyranoside			[52]	
			luteolin 7-*O*-β-D-glucuronopyranoside			[52]	
			luteolin 7-*O*-rutinoside			[52]	
			12-*O*-demethylcryptojaponol	AChE inhibitory (human erythrocytes)	50.8 μM	[52]	
				BChE inhibitory (horse serum)	70.1 μM	[52]	
			6α-hydroxydemethylcryptojaponol	AChE inhibitory (human erythrocytes)	19.2 μM	[52]	
				AChE inhibitory (electric eel)	12.3 μM	[52]	
				BChE inhibitory (horse serum)	7.70 μM	[52]	
			acteoside			[52]	
			isoacteoside			[52]	
			martynoside			[52]	
			stachysoside			[52]	
			incanide B			[52]	
	Caryopteris mongolica Bunge	roots	caryopteron A	antibacterial (*Staphylococcus aureus*)	12.9 mm, 5 μg/disc^{b}	[42]	**22**
				antibacterial (*Staphylococcus epidermidis*)	15.9 mm, 5 μg/disc^{b}	[42]	
				antibacterial (*Enterococcus faecalis*)	23.2 mm, 5 μg/disc^{b}	[42]	
				antibacterial (*Micrococcus luteus*)	16.3 mm, 5 μg/disc^{b}	[42]	
			caryopteron B			[42]	
			caryopteron C			[42]	

		caryopteron D			[42]	
		demethylcryptojaponol	antibacterial (*Staphylococcus aureus*)	11.8 mm, 50 μg/disc[b]	[42]	**23**
			antibacterial (*Staphylococcus epidermidis*)	13.7 mm, 50 μg/disc[b]	[42]	
			antibacterial (*Enterococcus faecalis*)	11.3 mm, 50 μg/disc[b]	[42]	
			antibacterial (*Micrococcus luteus*)	9.0 mm, 50 μg/disc[b]	[42]	
		6α-hydroxydemethyl cryptojaponol	antibacterial (*Staphylococcus aureus*)	11.0 mm, 50 μg/disc[b]	[42]	**24**
			antibacterial (*Staphylococcus epidermidis*)	11.5 mm, 50 μg/disc[b]	[42]	
			antibacterial (*Enterococcus faecalis*)	13.8 mm, 50 μg/disc[b]	[42]	
			antibacterial (*Micrococcus luteus*)	9.1 mm, 50 μg/disc[b]	[42]	
		deoxycoleon U	antibacterial (*Staphylococcus aureus*)	10.2 mm, 50 μg/disc[b]	[42]	
			antibacterial (*Staphylococcus epidermidis*)	10.2 mm, 50 μg/disc[b]	[42]	
			antibacterial (*Enterococcus faecalis*)	10.5 mm, 50 μg/disc[b]	[42]	
			antibacterial (*Micrococcus luteus*)	9.0 mm, 50 μg/disc[b]	[42]	
		(5*R*,10*S*,16*R*)-11,16-dihydroxy-12-methoxy-17(15→16)-abeo-abieta-8,11,13-trien -3,7-dione			[53]	
		(5*S*,10*S*,16*R*)-11,16-dihydroxy-12-methoxy-17(15→16)-abeo-abieta-8,11,13-trien -7-one	AChE inhibitory (human erythrocytes)	27.9 μM	[53]	**25**
			BChE inhibitory (horse serum)	73.8 μM	[53]	
		(5*R*,10*S*,16*R*)-11,12,16-trihydroxy-17(15→16)-abeo-abieta-8,11,13-trien-3,7-dione			[53]	
		(5*S*,10*S*,16*R*)-11,12,16-trihydroxy-17(15→16)-abeo-abieta-8,11,13-trien-7-one	AChE inhibitory (human erythrocytes)	64.6 μM	[53]	**26**
		(3*S*,5*R*,10*S*)-3,11,12,16-tetrahydroxy-17(15→16)-abeo-abieta-8,11,13-trien-7-one			[53]	
		(5*S*,10*S*,16*R*)-11,12,16-trihydroxy-17(15→16),18(4→3)-diabeo-abieta -3,8,11,13-tetraen-7-one	AChE inhibitory (human erythrocytes)	38.4 μM	[53]	**27**
			BChE inhibitory (horse serum)	98.4 μM	[53]	
		(5*S*,10*S*,16*R*)-11,12-dihydroxy-16-methoxy-17(15→16),18(4→3)-diabeo-abieta -3,8,11,13 -tetraen-7-one	AChE inhibitory (human erythrocytes)	20.8 μM	[53]	**28**
		(5*S*,10*S*,16*R*)-11,12,16,18-tetrahydroxy-17(15→16),18(4→3)-diabeo-abieta -3,8,11,13 -tetraen-7-one			[53]	
		(5*S*,10*S*,11*S*,12*S*,16*R*)-11,12,16-trihydroxy-17(15→16),18(4→3)-diabeo-abieta -3,8,13-trien-7-one			[53]	
		3,4-epoxy-11,12,16-trihydroxy-17(15→16),18(4→3)-diabeo -abieta-5,8,11,13-tetraen-7-one			[53]	
		(3*R*,5*R*,10*S*,16*R*)-3,11,12,16-tetrahydroxy-17(15→16),18(4→3)-diabeo-abieta -4(19),8,11,13-tetraen-7-one			[53]	
		(15*R*)-cyrtophyllone B	AChE inhibitory (human erythrocytes)	55.7 μM	[53]	**29**
			BChE inhibitory (horse serum)	93.2 μM	[53]	
		incanone	AChE inhibitory (human erythrocytes)	87.5 μM	[53]	**30**
Dracocephalum foetidum Bunge	aerial parts	2-[4-methyl-1-cyclohex-3-enyl]prop-2-en-1-*O*-6-malonyl-β-D-glucopyranosyl-(1→2)-6-malonyl-β-D-glucopyranoside			[47]	
		2-[4-methyl-1-cyclohex-3-enyl]prop-2-en-1-*O*-β-D-glucopyranosyl-(1→2)-6-malonyl-β-D-glucopyranoside			[47]	
		2-[4-methyl-1-cyclohex-3-enyl]prop-2-en-1-*O*-6-malonyl-β-D-glucopyranosyl-(1→2)-β-D-glucopyranoside			[47]	
		limonene-10-ol 10-*O*-β-D-glucopyranoside			[47]	
		4-*O*-(8-*Z*-isoferuloyl)-rosmarinic acid	DPPH radical-scavenging	30.5 μM	[47]	**33**
			hyaluronidase inhibitory	220 μM	[47]	
		rosmarinic acid	DPPH radical-scavenging	6.8 μM	[47]	**32**
			hyaluronidase inhibitory	750 μM	[47]	
		3'-*O*-methyl-rosmarinic acid			[47]	
		3-(6-malonyl-β-D-glucopyranosyl)-rosmarinic acid			[47]	
		3-(6-feruloyl-β-D-glucopyranosyl)-rosmarinic acid	DPPH radical-scavenging	13.2 μM	[47]	
		3-(6-synapoyl-β-D-glucopyranosyl)-rosmarinic acid			[47]	
		rosmarinic acid-3-*O*-β-D-glucopyranoside	DPPH radical-scavenging	31.6 μM	[47]	
		3-*O*-β-D-glucopyranosyl-7,8-*cis*-rosmarinic acid	DPPH radical-scavenging	16.4 μM	[47]	
		acacetin-7-*O*-(3-*O*-malonyl)-β-D-glucopyranoside			[47]	
		acacetin-7-*O*-(3,6-*O*-dimalonyl)-β-D-glucopyranoside	hyaluronidase inhibitory	250 μM	[47]	
		acacetin-7-*O*-(2-*O*-acetyl)-β-D-glucuronopyranoside			[47]	

		acacetin-7-*O*-(2-*O*-malonyl)-β-D-glucuronopyranoside			[47]
		acacetin-7-*O*-(3-*O*-malonyl)-β-D-glucuronopyranoside	hyaluronidase inhibitory	190 μM	[47]
		acacetin-7-*O*-β-D-glucopyranoside	DPPH radical-scavenging	204 μM	[47]
		acacetin-7-*O*-(6''-malonyl)-β-D-glucopyranoside			[47]
		acacetin-7-*O*-α-L-rhamnopyranosyl-(1-6)-β-D-glucopyranoside			[47]
		acacetin-7-*O*-β-D-glucuronide	hyaluronidase inhibitory	550 μM	[47]
		apigenin-7-*O*-(6''-malonyl)-β-D-glucopyranoside	DPPH radical-scavenging	156 μM	[47]
			hyaluronidase inhibitory	990 μM	[47]
		apigenin-7-*O*-β-D-glucuronide	DPPH radical-scavenging	63.2 μM	[47]
			hyaluronidase inhibitory	560 μM	[47]
		luteolin-7-*O*-β-D-glucuronide	DPPH radical-scavenging	19.8 μM	[47]
			hyaluronidase inhibitory	790 μM	[47]
		diosmetin-7-*O*-β-D-glucuronide	DPPH radical-scavenging	223 μM	[47]
		apigenin			[47]
Dracocephalum ruyschiana L.	aerial parts	acacetin-7-*O*-β-D-glucopyranosyl-(1→2)-β-D-glucopyranosyl-(1→2)-[α-L-rhamnopyranosyl-(1→6)]-β-D-glucopyranoside			[50]
		acacetin-7-*O*-β-D-glucopyranosyl-(1→2)-3-*O*-acetyl-β-D-glucopyranosyl-(1→2)-[α-L-rhamnopyranosyl-(1→6)]-β-D-glucopyranoside			[50]
		acacetin-7-*O*-β-D-glucopyranosyl-(1→2)-6-*O*-acetyl-β-D-glucopyranosyl-(1→2)-[α-L-rhamnopyranosyl-(1→6)]-β-D-glucopyranoside			[50]
		diosmetin-7-*O*-β-D-glucopyranosyl-(1→2)-6-*O*-acetyl-β-D-glucopyranosyl-(1→2)-[α-L-rhamnopyranosyl-(1→6)]-β-D-glucopyranoside			[50]
		apigenin-7-*O*-β-D-glucopyranosyl-(1→2)-β-D-glucopyranosyl-(1→2)-[α-L-rhamnopyranosyl-(1→6)]-β-D-glucopyranoside			[50]
		diosmetin 7-*O*-β-D-glucopyranosyl-(1→2)-β-D-glucopyranosyl-(1→2)-[α-L-rhamnopyranosyl-(1→6)]-β-D-glucopyranoside			[50]
		benzyl-2-*O*-*trans*-*p*-coumaroyl-α-L-rhamnopyranosyl-(1→6)-β-D-glucopyranoside			[50]
		benzyl-2-*O*-*cis*-*p*-coumaroyl-α-L-rhamnopyranosyl-(1→6)-β-D-glucopyranoside			[50]
		benzyl-4-*O*-*trans*-*p*-coumaroyl-α-L-rhamnopyranosyl-(1→6)-β-D-glucopyranoside			[50]
		benzyl-*O*-α-L-rhamnopyranosyl-(1→6)-β-D-glucopyranoside			[50]
		benzyl-*O*-β-D-glucopyranoside	DPPH radical-scavenging	223 μM	[50]
		[(benzoxy)methyl]phenyl-4-*O*-α-L-rhamnopyranosyl-(1→6)-β-D-glucopyranoside			[50]
		[(3-hydroxy-3-methylglutaryl)methyl]phenyl-4-*O*-β-D-glucopyranoside			[50]
		kaempferol 3-*O*-β-D-glucopyranoside			[50]
		quercetin 3-*O*-β-D-glucopyranoside	DPPH radical-scavenging	60.6 μM	[50]
		quercetin 3-*O*-β-D-glucuronide	DPPH radical-scavenging	37.2 μM	[50]
		chlorogenic acid	DPPH radical-scavenging	45.5 μM	[50]
		3,5-dicaffeoylquinic acid	DPPH radical-scavenging	32.4 μM	[50]
		3,4-dicaffeoylquinic acid	DPPH radical-scavenging	28.9 μM	[50]
		3-*p*-(*E*)-coumaroyl-5-(*E*)-caffeoylquinic acid	DPPH radical-scavenging	57.4 μM	[50]
		(7*S*,8*R*)-dihydrodehydrodiconiferyl alcohol-9'-*O*-β-D-glucopyranoside	DPPH radical-scavenging	284 μM	[50]
		(7*S*,8*R*)-urolignoside			[50]
		citrusin C	DPPH radical-scavenging	328 μM	[50]
		trans-*p*-coumaric acid			[50]
		methyl *trans*-*p*-coumaric acid	DPPH radical-scavenging	62.4 μM	[50]
		trans-ferulic acid			[50]
		cis-*p*-coumaric acid			[50]
		p-hydroxybenzaldehyde			[50]
		4,4'-dihydroxydiphenylmethane			[50]
Thymus gobicus Czern.	aerial parts	rosmarinic acid			[54]
		monardic acid A	AChE inhibitory (human erythrocytes)	4.0 μM	[54]
			AChE inhibitory (electric eel)	53.1 μM	[54]

		nepetoidin B	AChE inhibitory (human erythrocytes)	56.6 µM	[54]	
			AChE inhibitory (electric eel)	37.6 µM	[54]	
			BChE inhibitory (horse serum)	94.7 µM	[54]	
		aromadendrin			[54]	
		apigenin			[54]	
		chrysoriol			[54]	
		apigenin 7-*O* -β-D-glucuronopyranoside			[54]	
		apigenin 7-*O* -β-D-glucuronopyranoside methyl ester			[54]	
Pinaceae						
Abies sibirica Ledeb.	leaves	3α-hydroxymariesia-7,14,25(27)-trien-23-oxo-26-oic acid			[51]	
		(25*R*)-3α-hydroxy-25-methoxy-23-oxo-mariesia-7,14-dien-26-oic acid			[51]	
		(25*R*)-3α-hydroxy-25-methoxy-23-oxo-mariesia-7,12-dien-26-oic acid			[51]	
		23-hydroxy-3-oxomariesia-7,14,24-trien-26,23-olide			[51]	
		23-hydroxy-3-oxomariesia-8(9),14,24-trien-26,23-olide	LDL anti-oxidative	198.6 µM	[51]	
		3α,23-dihydroxylanosta-9(11),16,24-trien-26,23-olide	LDL anti-oxidative	55.3 µM	[51]	
		23-hydroxy-8(14→13)-abeo-17,13-fried-3-oxolanosta-8,14(15),24-triene-26,23- olide	LDL anti-oxidative	64.1 µM	[51]	
		(5*R* ,20*R*)-23-hydroxy-8(14→13*R*)-abeo-17,13-friedo-3-oxolanosta-8,14(30),24-triene-26,23-olide			[51]	
		(3α,9β,17α)-3,23,23-trihydroxy-17-methyl-γ-lactone-18-nordammara-7,12,24-trien-26-oic acid	LDL anti-oxidative	49.1 µM	[51]	
		7,14,24-mariesatrien-26,23-olide-3α,23-diol	mouse lipase inhibitory	510 µM	[51]	
			LDL anti-oxidative	39.4 µM	[51]	
		23-hydroxy-3-oxo-9β-lanosta-7,24-dien-26,23-olide	mouse lipase inhibitory	270 µM	[51]	
		23-hydroxy-3-oxolanosta-8,24-dien-26,23-olide			[51]	
		3,4-seco-4(28),6,8(14),24-mariesatetraene-26,23-olide-23-hydroxy-3-oic acid			[51]	
		23-oxo-mariesiic acid A			[51]	
		23-oxo-mariesiic acid B			[51]	
		(23*R* ,24*Z*) 3-oxo-9β-lanosta-7,24-dien-23-hydroxy-27-oic acid			[51]	
		13,17-*friedo* -3α-hydroxy-9β-lanosta-7,12,25(27)-trien-23-oxo-26-oic acid			[51]	
		isopseudolarifuroic acid B			[51]	
		dehydroabietic acid			[51]	
		bornyl acetate			[51]	
Polygonaceae						
Atraphaxis frutescens (L.) K. Koch	aerial parts	8-β-D-glucopyranosyloxy-3',4',5,5'-tetrahydroxy-7-methoxy-3-α-L-rhamnopyranosyloxyflavone	DPPH radical-scavenging	26.2 µM	[45]	**38**
			mushroom tyrosinase inhibitory	0.9 mM	[45]	
		8-acetoxy-3',4',5,5'-tetrahydroxy-7-methoxy-3-α-L-rhamnopyranosyloxyflavone	DPPH radical-scavenging	12.9 µM	[45]	**39**
			mushroom tyrosinase inhibitory	4.7 mM	[45]	
		3',4',5,5',8-pentahydroxy-7-methoxy-3-α-L-rhamnopyranosyloxyflavone	DPPH radical-scavenging	9.9 µM	[45]	**40**
			mushroom tyrosinase inhibitory	1.2 mM	[45]	
		8-acetoxy-3,3',4',5,5'-pentahydroxy-7-methoxyflavone	DPPH radical-scavenging	13.6 µM	[45]	
		3,3',4',5,5',8-hexahydroxy-7-methoxyflavone	DPPH radical-scavenging	15.4 µM	[45]	
		europetin 3-*O* -α-L-rhamnopyranoside	DPPH radical-scavenging	23.2 µM	[45]	**41**
			mushroom tyrosinase inhibitory	1.1 mM	[45]	**42**
		myricitrin	DPPH radical-scavenging	19.8 µM	[45]	
			mushroom tyrosinase inhibitory	2.0 mM	[45]	
		8-*O* -β-D-glucopyranosyl-7-*O* -methyl-3-*O* -α-L-rhamnopyranosylgossypetin	DPPH radical-scavenging	9.5 µM	[45]	
			mushroom tyrosinase inhibitory	4.0 mM	[45]	
		8-*O* -acetyl-7-*O* -methyl-3-*O* -α-L-rhamnopyranosylgossypetin	DPPH radical-scavenging	16.0 µM	[45]	
		7-*O* -methyl-3-*O* -α-L-rhamnopyranosylgossypetin	DPPH radical-scavenging	7.8 µM	[45]	

			mushroom tyrosinase inhibitory	2.2 mM	[45]	
		8-*O*-acetyl-7-*O*-methylgossypetin	DPPH radical-scavenging	11.2 μM	[45]	
		7-*O*-methylgossypetin	DPPH radical-scavenging	13.9 μM	[45]	
		fisetinidol 3'-*O*-β-D-glucopyranoside			[45]	
		gallocatechin	DPPH radical-scavenging	31.3 μM	[45]	**18**
			insect phenoloxidase inhibitory	37.5 μM	[45]	
			mushroom tyrosinase inhibitory	0.16 mM	[45]	
		afzelechin	DPPH radical-scavenging	139 μM	[45]	
			insect phenoloxidase inhibitory	230 μM	[45]	
			mushroom tyrosinase inhibitory	2.2 mM	[45]	
		epigallocatechin	DPPH radical-scavenging	23.6 μM	[45]	**20**
			insect phenoloxidase inhibitory	83.8 μM	[45]	
			mushroom tyrosinase inhibitory	0.7 mM	[45]	
		epicatechin	DPPH radical-scavenging	15.0 μM	[45]	
			insect phenoloxidase inhibitory	176 μM	[45]	
		3,4,5-trimethoxybenzyl α-L-rhamnopyranosyl-(1→6)-β-D-glucopyranoside	DPPH radical-scavenging	74.1 μM	[45]	
		nikoenoside	DPPH radical-scavenging	111 μM	[45]	
		N-*trans*-feruloyldopamine	DPPH radical-scavenging	30.7 μM	[45]	
			insect phenoloxidase inhibitory	291 μM	[45]	
		N-*trans*-feruloyltyramine	DPPH radical-scavenging	41.2 μM	[45]	
			insect phenoloxidase inhibitory	162 μM	[45]	
		N-*p*-*trans*-coumaroyldopamine	DPPH radical-scavenging	72.1 μM	[45]	
			insect phenoloxidase inhibitory	92.8 μM	[45]	
			mushroom tyrosinase inhibitory	4.3 mM	[45]	
		N-*p*-*trans*-coumaroyltyramine	insect phenoloxidase inhibitory	92.4 μM	[45]	
		fisetinidol			[45]	
		catechin			[45]	
		aromadendrin			[45]	
		emodin 8-*O*-β-D-glucopyranoside			[45]	
		emodin 8-*O*-(6'-*O*-malonyl)glucoside			[45]	
		torachrysone 8-*O*-β-D-(6'-*O*-malonyl)glucopyranoside			[45]	
		syringaresinol			[45]	
		dehydroconiferyl alcohol			[45]	
		3,4,5-trimethoxyphenyl 1-*O*-β-D-glucopyranoside			[45]	
		methyl syringate			[45]	
Calligonum mongolicum Turcz.	aerial parts	(*R*)-4-(4-hydroxyphenyl)-2-butanol 2-*O*-(6-*O*-galloyl)-β-D-glucopyranoside			[46]	
		5-(4-hydroxyphenyl) 2-pentenoic acid			[46]	
		(2*R*, 4a*S*, 8a*S*)-4a-hydroxy-2-methyl-3,4,4a,8a-tetrahydrobenzo-1 (2H)-pyran-7 (8)-one			[46]	
		rhododendrin			[46]	
		(*R*)-(−)-rhododendrol			[46]	
		4-(2-oxobutyl)phenol			[46]	
		catechin	insect phenoloxidase inhibitory	9.1 μM	[46]	**19**
		epicatechin	insect phenoloxidase inhibitory	148.3 μM	[46]	**21**
		dihydrokaempferol			[46]	
		dihydroquercetin			[46]	
		kaempferol			[46]	

		quercetin			[46]
		kaempferol 3-*O*-β-D-glucuronopyranoside			[46]
		quercetin 3-*O*-β-D-glucuronopyranoside			[46]
		N-*trans*-feruloyltyramine			[46]
		N-*cis*-feruloyltyramine			[46]
		2-(4-hydroxyphenyl)ethyl-β-D-glucopyranoside			[46]
		isopentyl β-D-glucopyranoside			[46]
		p-hydroxy-*trans*-cinnamic acid			[46]
		p-hydroxybenzoic acid			[46]
		protocatechuic acid			[46]
		gallic acid			[46]
Ranunculaceae					
Pulsatilla flavescens (Zucc.) Juz.	flowers	kaempferol	inhibition against *Babesia caballi*	220 μM	[40]
			inhibition against *Theileria equi*	201 μM	[40]
		apigenin 7-*O*-(6''-*O*-*p*-coumaroyl)-β-D-glucopyranoside	inhibition against *Babesia caballi*	18.8 μM	[40]
			inhibition against *Theileria equi*	55.6 μM	[40]
		kaempferol 3-*O*-(6''-*O*-*p*-coumaroyl)-β-D-glucopyranoside	inhibition against *Babesia caballi*	91.7 μM	[40]
			inhibition against *Theileria equi*	99.9 μM	[40]
		kaempferol 7-*O*-(6''-*O*-*p*-coumaroyl)-β-D-glucopyranoside	inhibition against *Babesia caballi*	26.2 μM	[40]
			inhibition against *Theileria equi*	76.0 μM	[40]
		apigenin 7-*O*-(6''-*O*-*p*-coumaroyl)-β-D-glucopyranoside	inhibition against *Babesia caballi*	57.0 μM	[40]
			inhibition against *Theileria equi*	45.2 μM	[40]
		apigenin 7-*O*-[6''-*O*-(3'''-hydroxy-3''-methylglutarate)]-β-D-glucopyranoside	inhibition against *Babesia caballi*	165 μM	[40]
			inhibition against *Theileria equi*	130 μM	[40]
		quercetin 3-*O*-β-D-glucopyranoside	inhibition against *Babesia caballi*	52.9 μM	[40]
			inhibition against *Theileria equi*	113 μM	[40]
		kaempferol 3-*O*-β-D-glucopyranoside	inhibition against *Babesia caballi*	135 μM	[40]
			inhibition against *Theileria equi*	222 μM	[40]
		quercetin 3-*O*-rutinoside	inhibition against *Babesia caballi*	94.3 μM	[40]
		kaempferol 3-*O*-rutinoside	inhibition against *Babesia caballi*	134 μM	[40]
			inhibition against *Theileria equi*	164 μM	[40]
		kaempferol 3-*O*-[6''-*O*-(*Z*)-*p*-coumaroyl]-β-D-glucopyranoside	inhibition against *Babesia caballi*	17.7 μM	[40]
			inhibition against *Theileria equi*	139 μM	[40]
		quercetin 3-*O*-β-D-glucuronopyranoside			[40]
		kaempferol 3-*O*-β-D-glucuronopyranosyl methyl ester			[40]
		chalconaringenin 2'-*O*-β-D-glucopyranosyl-5'-β-D-glucopyranoside			[40]
		(2*R*)-naringenin 8-β-D-glucopyranosyl-4'-*O*-β-D-glucopyranoside			[40]
		(2*S*)-naringenin 8-β-D-glucopyranosyl-4'-*O*-β-D-glucopyranoside			[40]
		(2*R*)-naringenin 6,8-di-β-D-glucopyranoside			[40]
		(2*S*)-naringenin 6,8-di-β-D-glucopyranoside			[40]
		(2*R*)-isohemipholin			[40]
		(2*S*)-isohemipholin			[40]
		naringenin 7-*O*-[6''-*O*-(*p*-coumaroyl)-β-D-glucopyranoside	inhibition against *Babesia caballi*	118 μM	[40]
			inhibition against *Theileria equi*	52.7 μM	[40]
Rosaceae					
Chamaerhodos altaica (Laxm.) Bunge	aerial parts	quercetin-3-*O*-β-D-glucuronopyranosyl-4'-*O*-β-D-glucopyranoside			[48]

		kaempferol-3-*O*-β-D-(6"-*O*-*trans*-*p*-coumaroyl)glucopyranoside	AGEs production inhibitory	260 μM	[48]
		kaempferol-3-*O*-β-D-(6"-*O*-*cis*-*p*-coumaroyl)glucopyranoside	DPPH radical-scavenging	266 μM	[48]
			AGEs production inhibitory	269 μM	[48]
		kaempferol-3-*O*-β-D-xylopyranosyl(1→2)-*O*-β-D-glucopyranoside			[48]
		quercetin-3-*O*-β-D-xylopyranosyl(1→2)-*O*-β-D-glucopyranoside	DPPH radical-scavenging	69.0 μM	[48]
		kaempferol-3-*O*-β-D-glucopyranosyl-7-*O*-β-D-glucuronopyranoside			[48]
		kaempferol-3-*O*-β-D-(6"-*O*-*trans*-*p*-coumaroyl)glucopyranosyl-7-*O*-β-D-glucopyranoside			[48]
		quercetin-3-*O*-β-D-glucuronide	DPPH radical-scavenging	24.3 μM	[48]
			AGEs production inhibitory	272 μM	[48]
		quercetin-3-*O*-β-D-glucopyranoside	DPPH radical-scavenging	13.4 μM	[48]
		kaempferol-3-*O*-β-D-glucopyranoside	DPPH radical-scavenging	230 μM	[48]
			AGEs production inhibitory	295 μM	[48]
		potentilin A	AGEs production inhibitory	166 μM	[48]
		sissotrin			[48]
		(+)-catechin	DPPH radical-scavenging	130 μM	[48]
			hyaluronidase inhibitory	842 μM	[48]
		(+)-catechin-7-*O*-β-D-glucopyranoside	DPPH radical-scavenging	95.8 μM	[48]
		tryptophan	DPPH radical-scavenging	656 μM	[48]
		lariciresinol-4'-β-D-glucopyranoside	DPPH radical-scavenging	332 μM	[48]
		trans-cinnamoyl-1-*O*-α-L-arabinopyranosyl-(1→6)-*O*-β-D-glucopyranoside			[48]
		benzyl-α-L-rhamnopyranosyl-(1→6)-*O*-β-D-glucopyranoside			[48]
		picein			[48]
		m-acetyl-β-D-glucopyranoside			[48]
		m-hydrocinnamoyl-β-D-glucopyranoside			[48]
		tachioside	DPPH radical-scavenging	198 μM	[48]
		shomaside F	DPPH radical-scavenging	34.6 μM	[48]
		strictinin	DPPH radical-scavenging	8.36 μM	[48]
		eugeniin	DPPH radical-scavenging	5.97 μM	[48]
			hyaluronidase inhibitory	509 μM	[48]
			AGEs production inhibitory	195 μM	[48]
		1,2,6-tri-*O*-galloyl-β-D-glucopyranoside	DPPH radical-scavenging	11.5 μM	[48]
			hyaluronidase inhibitory	792 μM	[48]
		potentillin	DPPH radical-scavenging	5.88 μM	[48]
			hyaluronidase inhibitory	890 μM	[48]
			AGEs production inhibitory	157 μM	[48]
		pedunculagin	DPPH radical-scavenging	9.18 μM	[48]
		agrimoniin	DPPH radical-scavenging	4.76 μM	[48]
			hyaluronidase inhibitory	578 μM	[48]
			AGEs production inhibitory	91.1 μM	[48]
Chamaerhodos erecta (L.) Bunge	aerial parts	4,5-dihydroxybenzaldehyde-3-*O*-β-D-glucopyranoside	DPPH radical-scavenging	400 μM	[48]
		kaempferol-3-*O*-β-D-(6"-*O*-*trans*-*p*-coumaroyl)glucopyranoside	AGEs production inhibitory	260 μM	[48]
		quercetin-3-*O*-β-D-glucuronide	DPPH radical-scavenging	24.3 μM	[48]
			AGEs production inhibitory	272 μM	[48]
		quercetin-3-*O*-β-D-glucopyranoside	DPPH radical-scavenging	13.4 μM	[48]
		kaempferol-3-*O*-β-D-glucopyranoside	DPPH radical-scavenging	230 μM	[48]
			AGEs production inhibitory	295 μM	[48]

		kaempferol-3-*O*-β-D-glucuronide			[48]
		quercetin-3-*O*-β-D-glucuronide methyl ether			[48]
		quercetin	DPPH radical-scavenging	65.2 μM	[48]
			AGEs production inhibitory	424 μM	[48]
		kaempferol	DPPH radical-scavenging	86.7 μM	[48]
			AGEs production inhibitory	381 μM	[48]
		tormentic acid			[48]
		euscaphic acid			[48]
		strictinin	DPPH radical-scavenging	8.36 μM	[48]
		1,2,3,4,6-penta-*O*-galloyl-β-D-glucopyranoside	DPPH radical-scavenging	5.50 μM	[48]
			hyaluronidase inhibitory	595 μM	[48]
			AGEs production inhibitory	230 μM	[48]
		eugeniin	DPPH radical-scavenging	5.97 μM	[48]
			hyaluronidase inhibitory	509 μM	[48]
			AGEs production inhibitory	195 μM	[48]
		casuarictin	DPPH radical-scavenging	6.41 μM	[48]
			AGEs production inhibitory	240 μM	[48]
		potentillin	DPPH radical-scavenging	5.88 μM	[48]
			hyaluronidase inhibitory	890 μM	[48]
			AGEs production inhibitory	157 μM	[48]
		agrimoniin	DPPH radical-scavenging	4.76 μM	[48]
			hyaluronidase inhibitory	578 μM	[48]
			AGEs production inhibitory	91.1 μM	[48]
		ellagic acid	DPPH radical-scavenging	22.0 μM	[48]
			AGEs production inhibitory	265 μM	[48]
Comarum salesovianum (Steph.) Aschers. Et Gr.	aerial parts	6-(non-8-enyl)salicylic acid	antibacterial (*Staphylococcus aureus*)	17.4 mm, 50 μg/disc[b]	[43]
			antibacterial (*Staphylococcus epidermidis*)	10.3 mm, 50 μg/disc[b]	[43]
			antibacterial (*Enterococcus faecalis*)	13.4 mm, 50 μg/disc[b]	[43]
			antibacterial (*Micrococcus luteus*)	18.8 mm, 50 μg/disc[b]	[43]
		6-nonyl salicylic acid	antibacterial (*Staphylococcus aureus*)	14.0 mm, 50 μg/disc[b]	[43]
			antibacterial (*Staphylococcus epidermidis*)	14.8 mm, 50 μg/disc[b]	[43]
			antibacterial (*Enterococcus faecalis*)	11.8 mm, 50 μg/disc[b]	[43]
			antibacterial (*Micrococcus luteus*)	16.6 mm, 50 μg/disc[b]	[43]
		3-(non-8-enyl) phenol	antibacterial (*Staphylococcus aureus*)	13.7 mm, 50 μg/disc[b]	[43]
			antibacterial (*Staphylococcus epidermidis*)	10.3 mm, 50 μg/disc[b]	[43]
			antibacterial (*Enterococcus faecalis*)	11.5 mm, 50 μg/disc[b]	[43]
			antibacterial (*Micrococcus luteus*)	29.9 mm, 50 μg/disc[b]	[43]
Dasiphora parvifolia (Fisch.. ex Lehm.) Juz.	aerial parts	5-hydroxy-7-*O*-(6-*O*-*p*-*cis*-coumaroyl-β-D-glucopyranosyl)-chromone			[49]
		5-hydroxy-7-*O*-(6-*O*-*p*-*trans*-coumaroyl-β-D-glucopyranosyl)-chromone			[49]
		ayanin 3'-*O*-β-D-glucopyranoside			[49]
		kaempferol-3-*O*-β-D-glucopyranoside			[49]
		quercetin-3-*O*-β-D-glucopyranoside			[49]
		quercetin-3-*O*-β-D-(6''-*O*-galloyl)glucopyranoside	DPPH radical-scavenging	24.4 μM	[49]
		quercetin-3-*O*-β-D-(6''-*O*-galloyl)galactopyranoside	DPPH radical-scavenging	17.7 μM	[49]
		kaempferol-3-*O*-β-D-(6''-*O*-*p*-*trans*-coumaroyl)glucopyranoside			[49]

		kaempferol-3-*O*-β-D-(6''-*O*-*p*-*cis*-coumaroyl)glucopyranoside			[49]	
		potentilin A			[49]	
		catechin-(7,8-bc)-4b-(3,4-dihydroxyphenyl)-dihydro-2(3*H*)-pyranone	DPPH radical-scavenging	18.5 μM	[49]	
			hyaluronidase inhibitory	750 μM	[49]	
		geranin A	DPPH radical-scavenging	336 μM	[49]	
			hyaluronidase inhibitory	822 μM	[49]	
		1,2,4,6-tetra-*O*-galloyl-β-D-glucopyranoside	DPPH radical-scavenging	6.7 μM	[49]	
		1,2,3,4,6-penta-*O*-galloyl-β-D-glucopyranoside			[49]	
Saxifragaceae						
Bergenia crassifolia (L.) Fritsch	roots	catechin			[39]	
		(−)-epicatechin-3-*O*-*p*-hydroxybenzoate			[39]	
		catechin-3-*O*-gallate			[39]	
		catechin-5-*O*-gallate			[39]	
		(2*R*,3*S*)-3-*O*-*p*-hydroxybenzoyl-5-*O*-galloylcatechin			[39]	
		catechin-3,5-di-*O*-gallate	inhibition against *Babesia bovis*	0.83 μg/mL	[39]	**14**
			inhibition against *Babesia bigemina*	5.69 μg/mL	[39]	
		catechin-3,7-di-*O*-gallate	inhibition against *Babesia bovis*	0.80 μg/mL	[39]	**15**
			inhibition against *Babesia bigemina*	6.31 μg/mL	[39]	
		(+)-afzelechin			[39]	
		kaemferol-3-*O*-β-D-galactopyranoside			[39]	
		kaemferol-3-*O*-glucoside			[39]	
		quercetin-3-*O*-β-D-glucopyranoside			[39]	
		rhamnazin			[39]	
		arbutin-4-*O*-*p*-hydroxybenzoyl			[39]	
		arbutin-6-*O*-gallate			[39]	
		6'-*O*-(3''-*O*-methylgalloyl) arbutin			[39]	
		arbutin-4,6-di-*O*-gallate	inhibition against *Babesia bovis*	8.24 μg/mL	[39]	
			inhibition against *Babesia bigemina*	6.59 μg/mL	[39]	
		glucose-1,2,6-tri-*O*-gallate	inhibition against *Babesia bovis*	10.3 μg/mL	[39]	
			inhibition against *Babesia bigemina*	6.41 μg/mL	[39]	
		bergenin			[39]	
		bergenin-11-*O*-*p*-hydroxybenzoyl			[39]	
		bergenin-4-*O*-gallate			[39]	
		bergenin-11-*O*-gallate			[39]	
		bergenin-4,11-di-*O*-gallate	inhibition against *Babesia bovis*	6.04 μg/mL	[39]	
			inhibition against *Babesia bigemina*	8.60 μg/mL	[39]	
Saxifraga spinulosa Adams	aerial parts	(2*S*)-3'-*O*-β-D-(6''-*O*-galloyl)glucopyranosyloxy-5,7,4',5'-tetrahydroxyflavanone	inhibition against *Babesia bovis*	9.4 μM	[38]	**11**
			inhibition against *Babesia bigemina*	19.9 μM	[38]	
			DPPH radical-scavenging	53.1 μM	[38]	
		(2*R*)-3'-*O*-β-D-(6''-*O*-galloyl)glucopyranosyloxy-5,7,4',5'-tetrahydroxyflavanone	inhibition against *Babesia bovis*	12.1 μM	[38]	**12**
			inhibition against *Babesia bigemina*	22.7 μM	[38]	
			DPPH radical-scavenging	58.8 μM	[38]	
		(2*S*)-3'-*O*-β-D-[6''-*O*-(3'''-*O*-methyl)galloyl]glucopyranosyloxy-5,7,4',5'-tetrahydroxyflavanone	DPPH radical-scavenging	64.9 μM	[38]	
		(2*S*)-3'-*O*-β-D-(2'',6''-di-*O*-galloyl)glucopyranosyloxy-5,7,4',5'-tetrahydroxyflavanone	DPPH radical-scavenging	42.3 μM	[38]	
		(2*R*,3*R*)-3'-*O*-β-D-(6''-*O*-galloyl)glucopyranosyloxy-5,7,4',5'-tetrahydroxyflavanonol	DPPH radical-scavenging	29.3 μM	[38]	
		(2*R*,3*R*)-3'-*O*-β-D-(2'',6''-di-*O*-galloyl)glucopyranosyloxy-5,7,4',5'-tetrahydroxyflavanonol	DPPH radical-scavenging	42.5 μM	[38]	

	inactivation (feline calicivirus)	1.5[c] (1 min, 25 μg/mL)	[41]	
3'-*O*-β-D-(6''-*O*-galloyl)glucopyranosyloxy-5,7,4',5'-tetrahydroxyflavonol	DPPH radical-scavenging	44.7 μM	[38]	
(2*S*)-3'-*O*-β-D-(6''-*O*-galloyl)glucopyranosyloxy-5,7,4'-trihydroxyflavanone	DPPH radical-scavenging	72.9 μM	[38]	
8-*O*-β-D-[6'-*O*-(3''-*O*-methyl)galloyl]glucopyranosyl-*p*-tyrosol	DPPH radical-scavenging	49.1 μM	[38]	
8-*O*-β-D-(2',6'-di-*O*-galloyl)glucopyranosyl-*p*-tyrosol	inhibition against *Babesia bovis*	5.9 μM	[38]	**13**
	inhibition against *Babesia bigemina*	27.1 μM	[38]	
	inhibition against *Babesia caballi*	15.6 μM	[38]	
	DPPH radical-scavenging	31.4 μM	[38]	
4-*O*-β-D-(6'-*O*-galloyl)glucopyranosyl-(*E*)-*p*-coumaroyl acid	DPPH radical-scavenging	35.1 μM	[38]	
3-*O*-β-D-(6'-galloyl)glucopyranosyl-5-*O*-methylgallic acid	DPPH radical-scavenging	19.0 μM	[38]	
gallocatechin-3-*O*-gallate	inhibition against *Babesia bovis*	3.8 μM	[38]	**16**
	inhibition against *Babesia bigemina*	11.0 μM	[38]	
	inactivation (human influenza A virus)	2.17[c] (1 min, 25 μg/mL)	[41]	
	inactivation (feline calicivirus)	2.5[c] (1 min, 25 μg/mL)	[41]	
	inactivation (mouse norovirus)	1.25[c] (6 hrs, 100 μg/mL)	[41]	
	inactivation (SARS-CoV-2)	1.7[c] (1 min, 25 μg/mL)	[41]	
myricetin	inhibition against *Babesia caballi*	51.3 μM	[38]	
	inhibition against *Theileria equi*	41.7 μM	[38]	
rutin	inhibition against *Babesia caballi*	20.3 μM	[38]	
quercetin 3-*O*-β-D-glucopyranoside	inhibition against *Babesia caballi*	23.7 μM	[38]	
quercetin 3-*O*-β-D-(6''-*O*-galloyl)glucopyranoside	inhibition against *Babesia bigemina*	27.2 μM	[38]	
	inhibition against *Babesia caballi*	9.7 μM	[38]	
quercetin 3-*O*-β-D-(6''-*O*-galloyl)galactopyranoside	inhibition against *Babesia caballi*	4.4 μM	[38]	
myricetin 3-*O*-β-D-galactopyranoside	inhibition against *Babesia caballi*	36.7 μM	[38]	
	inhibition against *Theileria equi*	40.6 μM	[38]	
eriodictyol	inhibition against *Babesia bigemina*	46.7 μM	[38]	
taxifolin	inhibition against *Babesia bigemina*	15.0 μM	[38]	
	inhibition against *Babesia caballi*	41.5 μM	[38]	
	inhibition against *Theileria equi*	40.6 μM	[38]	
4-(4'-hydroxyphenyl)-2-butanone 4'-*O*-β-D-(2'',6''-di-*O*-galloyl)glucopyranoside	inhibition against *Babesia bovis*	6.4 μM	[38]	
	inhibition against *Babesia bigemina*	15.3 μM	[38]	
	inhibition against *Babesia caballi*	2.5 μM	[38]	
gallocatechin			[38]	
catechin			[38]	
epigallocatechin-3-*O*-gallate	inactivation (human influenza A virus)	1.17[c] (1 min, 25 μg/mL)	[41]	**17**
	inactivation (feline calicivirus)	2.5[c] (1 min, 25 μg/mL)	[41]	
	inactivation (mouse norovirus)	1.5[c] (6 hrs, 100 μg/mL)	[41]	
	inactivation (SARS-CoV-2)	1[c] (1 min, 25 μg/mL)	[41]	
3-*O*-(6''-*O*-galloyl-β-D-glucopyranosyl)gallocatechin			[38]	
quercetin			[38]	
luteolin			[38]	
quercetin 3-*O*-α-L-rhamnopyranoside			[38]	
dihydromyricetin	inactivation (human influenza A virus)	1.83[c] (1 min, 25 μg/mL)	[41]	
	inactivation (feline calicivirus)	2[c] (1 min, 25 μg/mL)	[41]	
(2*R*,3*R*)-dihydromyricetin 3'-*O*-β-D-glucopyranoside	inactivation (feline calicivirus)	1.88[c] (1 min, 25 μg/mL)	[41]	

			inactivation (human influenza A virus)	2[c] (6 hrs, 25 μg/mL)	[41]
		naringenin 7-*O*-β-D-(6''-*O*-galloyl)glucopyranoside			[38]
		tyrosol			[38]
		6''-*O*-galloyl salidroside	inactivation (feline calicivirus)	2.33[c] (1 min, 25 μg/mL)	[41]
			inactivation (human influenza A virus)	1.5[c] (6 hrs, 25 μg/mL)	[41]
		phenethyl *O*-α-L-rhamnopyranosyl-(1-6)-*O*-β-D-glucopyranoside			[38]
		gallic acid	inactivation (feline calicivirus)	1.33[c] (1 min, 25 μg/mL)	[41]
Polyporaceae					
Fomitopsis officinalis Vill. ex Fr. Bond	fruitng bodies	fomitopsin G			[36]
		fomitopsin H	inhibition against *Trypanosoma congolense*	27.1 μM	[36]
		demalonyl fomitopsin H	inhibition against *Trypanosoma congolense*	12.5 μM	[36]
		fomitopsin D ethyl ester	inhibition against *Trypanosoma congolense*	15.0 μM	[36]
		fomitopsin F	inhibition against *Trypanosoma congolense*	26.0 μM	[36]
		(25*S*)-(+)-12α-hydroxy-3α-malonyloxy-24-methyllanosta-8,24(31)-dien-26-oic acid			[36]
		fomefficinic acid G			[36]
		15α-hydroxy-3-oxo-24-methylenelanosta-7,9(11)-dien-21-oic acid	inhibition against *Trypanosoma congolense*	7.0 μM	[36]

[a] There are exceptions. [b] The inhibition zone diameter. [c] $\log_{10}TCID_{50}$/mL.
